# Nanosilver induces minimal lung toxicity or inflammation in a subacute murine inhalation model

**DOI:** 10.1186/1743-8977-8-5

**Published:** 2011-01-25

**Authors:** Larissa V Stebounova, Andrea Adamcakova-Dodd, Jong Sung Kim, Heaweon Park, Patrick T O'Shaughnessy, Vicki H Grassian, Peter S Thorne

**Affiliations:** 1 Department of Chemistry, University of Iowa, Iowa City, IA 52242, USA; 2 Department of Occupational and Environmental Health, University of Iowa, Iowa City, IA 52242, USA; 3 Interdisciplinary Graduate Program in Human Toxicology, University of Iowa, Iowa City, IA 52242, USA

## Abstract

**Background:**

There is increasing interest in the environmental and health consequences of silver nanoparticles as the use of this material becomes widespread. Although human exposure to nanosilver is increasing, only a few studies address possible toxic effect of inhaled nanosilver. The objective of this study was to determine whether very small commercially available nanosilver induces pulmonary toxicity in mice following inhalation exposure.

**Results:**

In this study, mice were exposed sub-acutely by inhalation to well-characterized nanosilver (3.3 mg/m^3^, 4 hours/day, 10 days, 5 ± 2 nm primary size). Toxicity was assessed by enumeration of total and differential cells, determination of total protein, lactate dehydrogenase activity and inflammatory cytokines in bronchoalveolar lavage fluid. Lungs were evaluated for histopathologic changes and the presence of silver. In contrast to published *in vitro *studies, minimal inflammatory response or toxicity was found following exposure to nanosilver in our *in vivo *study. The median retained dose of nanosilver in the lungs measured by inductively coupled plasma - optical emission spectroscopy (ICP-OES) was 31 μg/g lung (dry weight) immediately after the final exposure, 10 μg/g following exposure and a 3-wk rest period and zero in sham-exposed controls. Dissolution studies showed that nanosilver did not dissolve in solutions mimicking the intracellular or extracellular milieu.

**Conclusions:**

Mice exposed to nanosilver showed minimal pulmonary inflammation or cytotoxicity following sub-acute exposures. However, longer term exposures with higher lung burdens of nanosilver are needed to ensure that there are no chronic effects and to evaluate possible translocation to other organs.

## Background

With increasing use of manufactured nanoparticles the potential for exposure among manufacturers and consumers is also increasing. Many of these manufactured nanomaterials are composed of metal and metal oxides and there have been reports of metal-containing nanoparticles in workplaces [1], from industrial sources detected in the atmosphere [2,3] and in surface waters [4,5]. Silver nanoparticles, often referred to as nanosilver, may be problematic because of their use in a wide range of applications due to their antimicrobial activity [6-8]. Nanosilver is used for treatments of wounds, burns, water or air disinfection, or as coatings on various textiles [9]. There is concern that little is known about the environmental and health consequences of exposure to nanosilver.

Prior *in vitro *studies of nanosilver toxicity [10-15] reveal toxic effects of nanosilver through reduced cell viability, damage to the cell membrane and other biological effects on the organism. Nanosilver has been reported to be among the most toxic nanomaterials in some studies [10,16-18]. The cytotoxicity of nanosilver has been associated with generation of reactive oxygen species [10,19] and several studies suggest that nanosilver is only toxic when oxidized to silver ions, Ag^+ ^[12,14].

Most of the *in vitro *studies show dose dependence, where higher doses of Ag induce higher cellular toxicity. In contrast to *in vivo *investigations, *in vitro *concentrations of nanoparticles are often much higher and the particles are delivered to the cells via the culture medium. Such exposures do not replicate the conditions expected for *in vivo *exposure. Currently, there is no direct correlation of biological markers of toxicity between *in vivo *and *in vitro *studies due to the complexity of dose delivery, mono- vs. co-cultures, endpoint evaluations as well as other factors of cellular interaction with different biological media [20,21]. Even though air-liquid *in vitro *systems have been recently developed that may have more predictive value [22-25], *in vivo *studies are still considered to be more relevant for risk assessment.

Although inhalation is considered the most important route of exposure for nanoparticles [26,27], little is known about the environmental and health risks of aerosolized nanosilver [28]. There are a few reports in the literature on pulmonary studies of nanosilver toxicity in rats [7,29-32]. In these studies silver was detected in the lungs and, at much lower concentration, in the liver, brain, olfactory bulb and blood indicating organ translocation of nanosilver. No significant health implications were found for short exposure time periods although some pathologic responses were observed for higher Ag doses or longer exposures, such as chronic alveolar inflammation, small granulomatous lesions, and a decrease in the tidal and minute volume of the lungs. However, there are significant gaps in the toxicity studies of nanosilver and more thorough investigations with well characterized materials are warranted [6].

An integrated approach that includes both inhalation toxicology studies and full characterization of the nanomaterials is necessary for understanding inflammatory responses as they relate to the physicochemical principles of nanoparticle toxicity. In the current study, such an approach is used to assess the potential toxic effects associated with the inhalation of commercial nanosilver using a murine model for lung inflammation, histopathology, and dosimetry.

### Materials and methods

#### Nanoparticles

Nanosilver with a manufacturer's stated average particle size of 10 nm (minimum 5 nm, maximum 15 nm, BET= (10 + 1) m^2^/g) were in powder form and used as received from the manufacturer (Stock # 0478 YD, Nanostructured and Amorphous Materials, Inc, Houston, TX).

The nanosilver was characterized using transmission electron microscopy (TEM), X-ray diffraction (XRD), X-ray photoelectron spectroscopy (XPS), and BET techniques [33]. The bulk crystalline phases of Ag nanoparticles were determined using powder XRD measurements (Bruker D-5000 q - q diffractometer with Kevex-sensitive detector, Madison, WI). TEM (JEOL JEM-1230, Japan) was used to measure the primary particle size of 500 random nanoparticles and compare the average to manufacturer's specifications.

Surface properties, surface area, and composition of the Ag nanoparticles were examined. Surface area measurements of the powder sample were made on automated multipoint BET surface area apparatus (Quantachrome Nova 4200e, Boynton Beach, Florida) using nitrogen as the adsorbent. X-ray photoelectron spectroscopy (XPS) was used to probe the surface chemical composition characteristics of the metal nanoparticles (custom-designed Ultra-Axis XPS system from Kratos, Manchester, UK).

Dissolution studies were conducted in two different types of artificial fluids using inductively coupled plasma optical emission spectrometer (ICP-OES) (Varian Inc. 720-ES, Walnut Creek, CA). Ag nanoparticles were incubated in artificial interstitial fluid (Gamble's solution, pH = 7.4) and artificial lysosomal fluid (ALF, pH = 4.5) at a concentration of 2 mg/ml for 24 hours at 38°C. The ALF solution simulates the composition and pH of alveolar and interstitial macrophages and the Gamble's solution simulates interstitial fluid in the lungs [34]. The compositions of the simulated biological fluids can be found in Ref. [35]. The final solutions were filtered by a syringe through 0.2 μm filter and centrifuged for 30 minutes at 14,000 rpm in order to remove nanoparticles and aggregates that were not dissolved. The filtered and centrifuged solutions were analyzed by ICP-OES.

#### Animals

Male (6 weeks old) C57Bl/6 mice (The Jackson Laboratory, Bar Harbor, ME) were quarantined for 12 days after their arrival in our vivarium in polypropylene, fiber-covered cages in HEPA-filtered Thoren caging units (Hazelton, PA) in the Pulmonary Toxicology Facility at the University of Iowa prior to use. Mice were supplied with food (sterile Teklad 5% stock diet, Harlan, Madison, WI) and water *ad libitum *and maintained on a 12-hr light-dark cycle. The average weight of the animals at the time of necropsy was 22.4 g at 0 wk post exposure and 26.8 g at 3 weeks post exposure. Sham-exposed control mice had a mean weight of 26.9 g at the time of necropsy. Protocols were approved by the Institutional Animal Care and Use Committee at the University of Iowa and complied with the NIH Guide for the Care and Use of Laboratory Animals.

#### Exposure Generation and Characterization

A dynamic whole-body exposure chamber [36] was used to expose animals to nanosilver. Sham-exposed mice breathed filtered room air in an identical chamber setup. A suspension of nanosilver in ultra-pure water (Milli-Q^® ^Academic A-10, Millipore Corp., Billerica, MA) was ultra-sonicated (model 550, Fisher Scientific, Pittsburgh, PA) for 20 min. Aerosols of this suspension were generated using a 6-jet Collison nebulizer (BGI Inc., Waltham, MA) supplied with filtered, pressurized air (24 psi) and passed through a 60 cm × 2 cm (I.D.), brass drying column heated to 110°C as well as a 20 mCi ^63^Ni source to completely evaporate droplets and neutralize charges on the particles prior to introduction into the exposure chamber. Gravimetric concentrations of particles were measured in the chamber using 47-mm glass microfiber filters (Whatman, Middlesex, United Kingdom) in line with the 24 l/min exhaust airflow. With the additional heat and humidity supplied to the chamber, the temperature and relative humidity in the exposure chamber during the experiments was controlled between 20-22°C and 25-35%, respectively. Electron microscopy grids were placed inside the exposure chamber for analysis of deposited nanoparticles. The grids were analyzed using scanning electron microscopy with an energy dispersive spectrometer (SEM-EDS) (Hitachi S-3400N, Japan) capable of providing elemental analysis of the particles. This confirmed the presence of nanosilver on the grid from the whole-body chamber during inhalation exposures. The size distribution of the aerosol in the whole-body exposure chamber was measured using a scanning mobility particle sizer (SMPS, TSI Inc., Shoreview, MN) for diameters in the range of 7.4 to 289 nm. Geometric mean (GM) and geometric standard deviation (GSD) of aerosol sizes in individual exposures were calculated from SMPS measurements.

#### Inhalation Exposure

**Figure 1:**
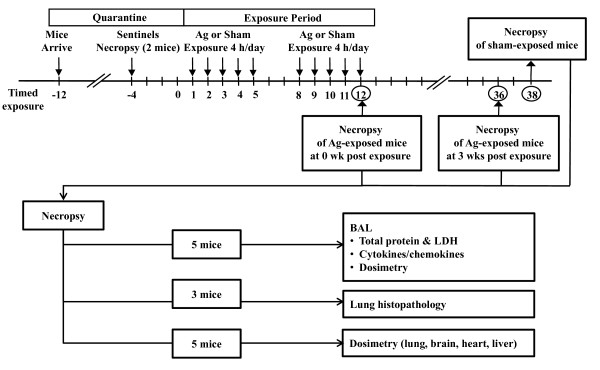
**Schematic of the experimental design used in this study**.

Mice were exposed sub-acutely to nanosilver for 4 hours/day, 5 days/week for 2 weeks and necropsied within one hour (0 wk) or 3 weeks post exposure (3 wks). The experimental design of this study is shown in Figure 1. The average concentration of nanosilver was 3.3 ± 0.5 mg/m^3 ^(range = 2.4 - 4.0 mg/m^3^). A minute volume of 25 ml and particle deposition fraction (α) in the tracheo-bronchiolar and pulmonary region of 0.15 [37,38] were assumed to estimate nanosilver dose.

#### Bronchoalveolar Lavage

Mice were euthanized by isoflurane inhalation and exsanguination through the heart. Lungs (from 5 animals per each experimental group) were lavaged 3 times with 1 ml of 0.9% sterile sodium chloride solution (Baxter, Deerfield, IL) and the cell pellet was used for enumeration of total and differential cell counts as previously described [39]. Cells recovered from bronchoalveolar lavage (BAL) fluid were processed for TEM-EDS analyses (n = 1). Macrophages recovered from BAL fluid with and without Ag particles (100 cells per sample) were counted using dark field microscopy to assess the extent of the phagocytosis. The lavage supernatants (n = 5) were analyzed for total protein in 4 dilutions (1:5, 1:10, 1:20, and 1:40) using a Bradford protein assay (Bio-Rad Laboratories, Inc., Hercules, CA), lactate dehydrogenase (LDH) activity was measured in duplicates with a commercial assay (Roche Diagnostics, Penzberg, Germany) and cytokine levels measured by multiplexed fluorescent bead-based immunoassays (Bio-Plex Pro Mouse Cytokine, Chemokine, and Growth Factor Multiplex Assays, Bio-Rad Laboratories, Inc., Hercules, CA). Measured cytokines included: interleukin (IL)-6, IL-12(p40), tumor necrosis factor (TNF)-α, granulocyte macrophage colony stimulating factor (GM-CSF), keratinocyte-derived cytokine (KC), monocyte chemotactic protein (MCP)-1, and macrophage inflammatory protein (MIP)-1α. The BAL fluid (n = 5) was then centrifuged again at 22,000 rpm for 30 min and Ag concentration in BAL supernatants was measured by ICP-MS.

#### Histopathology

Lungs from non-lavaged mice (n = 3) were fixed in 10% formaldehyde-phosphate-buffered saline solution via the canulated trachea. The tissues were paraffin-embedded, sectioned at 5 μm, and stained with hematoxylin and eosin (H & E) as previously described [25]. Tissue sections were evaluated by light microscopy to elucidate abnormalities of the parenchymal architecture (bronchioles, alveoli, pleura, and vasculature); abnormal inflammatory infiltrates; presence or absence of acute lung injury; and presence or absence of fibrosis.

#### Nanosilver dosimetry

Lung, brain, heart, and liver tissues from 5 mice in each experimental group were stored at -80°C immediately after resection. Tissues were lyophilized for 16 hours at 1.3 × 10^-1 ^mBar and -50°C in a freeze dryer (Labconco Corp., Kansas City, MO) and then weighted. High purity concentrated nitric acid (Fisher Optima^® ^grade) was used to digest the tissues at 95-98°C. Metal analysis was performed using ICP-OES (Varian 720 ES, Varian Inc., Walnut Creek, CA) with a method detection limit for Ag of 1.8 μg/g lung dry weight (d.w.).

#### EDS-spectra of Ag nanoparticles in alveolar macrophages

The BAL cells were fixed with 2.5% glutaraldehyde in 0.1M Na cacodylate buffer, post fixed with 1% osmium tetroxide, dehydrated through graded ethanols and embedded in epoxy resin. Thins sections were cut at ~80-90 nm on a Leica EM UC6 ultramicrotome (Leica Microsystems GmbH, Wetzlar, Germany) and placed on 200 mesh formvar/carbon coated nickel grids. Elemental analysis of the cells recovered from BAL was performed with a Thermo Fisher Noran System 7 (Waltham, MA) energy dispersive spectroscopy system (EDS) attached to a JOEL JEM-2100F (Tokyo, Japan) field emission transmission electron microscope. The TEM was operated at an accelerating voltage of 200 kV in scanning mode combined with a high angle annular field detector (HAADF). NSS 2.2 software package was used to acquire and process the data.

#### Statistical Analyses

Data from nanosilver-exposed and sham-exposed animals were compared using t tests for equal or unequal variances (SAS Ver. 9.2, SAS, Inc., Cary, NC). A p-value less than 0.05 was considered significant. Data are expressed as mean ± standard error (SE) unless otherwise noted.

## Results

### Particle Characterization

**Figure 2:**
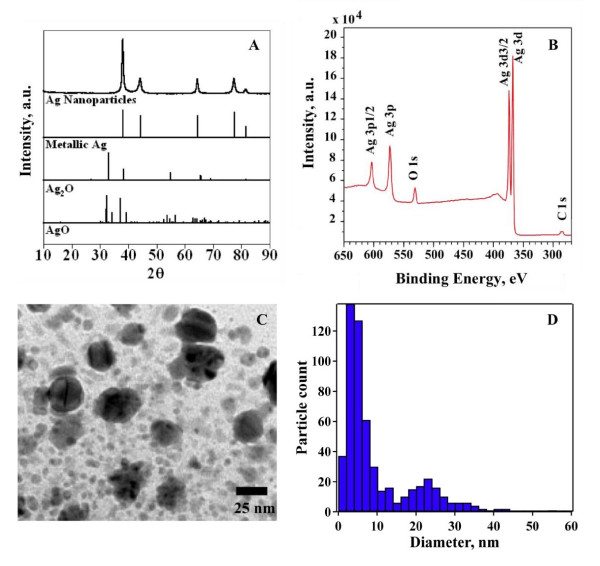
**A. Powder X-ray diffraction patterns for nanosilver with patterns shown for three reference compounds including Ag, AgO and AgO**_**2**_. B. X-ray photoelectron spectrum for nanosilver. C. TEM image of nanosilver and D. particle primary diameter count distribution from TEM images.

The results of particle characterization are summarized in Figure 2 and Table 1. The XRD patterns in Figure 2A compare the nanosilver results with metallic silver and silver oxide (Ag_2_O, AgO) reference spectra and demonstrate that the nanoparticles are metallic silver with no detectable silver oxide. Surface composition was also examined using XPS (Figure 2B) to test for the presence of an oxide surface layer. Peaks in the Ag3d, Ag3p, O1s, and C1s regions of the photoelectron spectrum were identified. The Ag3d doublet at 368.2 eV and 374.2 eV is consistent with silver in the Ag^0 ^oxidation state. The O1s region does not show any oxygen peaks below 530 eV attributable to AgO or Ag_2_O, but there is a peak due to CO_3_^2- ^at 530.7 eV. There are also unique peaks at 287.3 and 288.7 eV which we attribute to C=O and CO_3_^2-^, respectively. In summary, the XPS data indicate the presence of adventitious carbon, some carbon-oxygen functionality, likely due to the use of polyvinylpyrrolidone (PVP) during synthesis to control particle size, and Ag_2_CO_3 _on the surface of the nanosilver, but no evidence of a silver oxide coating. This coating accounts for up to 17% of the nanoparticle mass, as determined by ICP analysis and corresponds to a coating thickness of approximately 0.7 nm. A presence of PVP on Ag nanoparticles surface diminishes the propensity of nanoparticles towards aggregation [40] and shows a minimal effect on the Ag nanoparticles toxicity against prokaryotic bacteria [12]. The specific surface area of nanosilver was determined to be 3 ± 2 m^2^/g using multi-point BET analysis and is lower than the manufacturer's specified specific surface area of 10 ± 1 m^2^/g.

**Table 1:** Summary of physicochemical characterization data of Ag nanoparticles and Ag nanoparticle aerosols

Primary Particle Size*	TEM	5 ± 2 nm; 22 ± 4 nm
Crystalline or Amorphous Material, phase	XRD	Crystalline, face-centered cubic, metal

Surface Functionality	XPS	CO_3 _^2-^, OH^-^, C=O, C-O-C

BET Surface Area	BET	3 ± 2 m^2^/g

Aerosol Concentration	Gravimetric Analysis	3.3 ± 0.5 mg/m^3^

Aerosol Size Distribution**	SMPS	79 nm (1.5)

* Bimodal distribution with smaller particles account for 85-90% of the total particle count.

**GM, nm, (GSD) in exposure chamber

A primary particle size distribution was generated from the TEM images (Figure 2C) by measuring the two-dimensional diameter of more than 500 nanoparticles (Figure 2D). The first mode of the bimodal particle size distribution with a peak maximum at 5 nm accounted for 85 to 90% of the total particle count. The second mode, with a peak at 22 nm, was attributed to larger particles that account for less than 15% of the total particle count.

**Figure 3:**
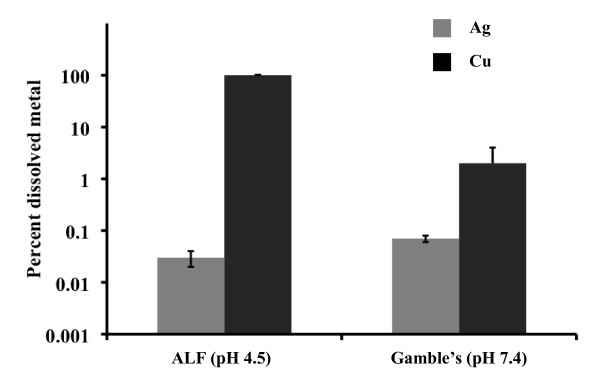
**Dissolution results for Ag and Cu nanoparticles in ALF and Gamble's solution after 24 hours at 38°C. Cu nanoparticle data are from Ref. 34**.

The dissolution of Ag nanoparticles into Ag ions in ALF and Gamble's solution was studied using ICP-OES [34]. The bioavailability and toxicity of metal nanoparticles are often linked to the ability of the nanoparticles to deliver soluble metal ions to the surrounding tissue. Figure 3 shows that nanosilver dissolved less than 0.1% in either simulated biological fluid in 24 hours. The concentrations of Ag ions were measured in triplicate in ALF and Gamble's buffers with the dissolved metal percentages of 0.03 ± 0.002% and 0.07 ± 0.003%, correspondingly. This is in contrast to earlier studies on Cu nanoparticles that fully dissolved in ALF and showed 2% dissolution in Gamble's solution [34].

**Figure 4:**
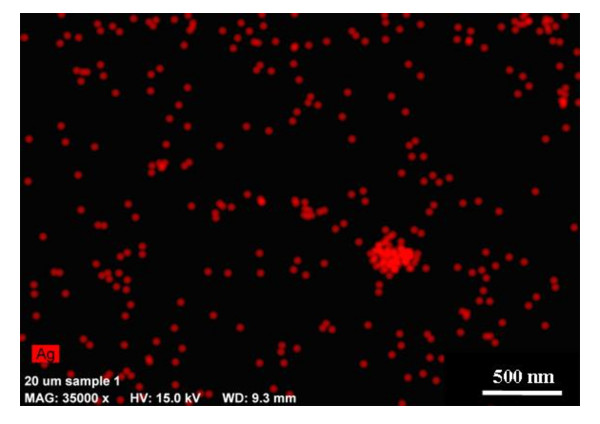
**SEM-EDS mapping of nanosilver aerosols deposited on TEM grid during the exposure**. The colored dots represent the elemental map for Ag at the expected characteristic X-ray energy for Ag and show, based on size, the presence of silver nanoparticle agglomerates.

SEM-EDS of the particles collected on TEM grids in the whole body chamber showed nanosilver aerosol between 50 and 100 nm detected at the characteristic X-ray energy for Ag (Figure 4). The size distributions of Ag nanoparticle aerosols measured during exposure using an SMPS show that the nanosilver aerosols have a GM mobility diameter of 79 nm and GSD of 1.5 (Table 1). These measurements show that mice were exposed to agglomerates of nanosilver that were larger than the primary nanoparticles.

### Sub-acute Exposure

The median amount of silver measured by ICP-OES in the lungs of nanosilver-exposed mice necropsied immediately after the exposure (0 wk group) was 31 μg/g lung (d.w.) (range 4.3 to 37.5 μg/g lung (d.w.)). This represents the loading in the lungs but not the nose, nasopharynx or trachea, which were not measured. The lungs of the 3 wk mice had a median silver content of 10 μg/g lung (d.w.), and two of these five mice had no detectable silver. The mass concentration of silver in all sham-exposed mice was below the detection limit (1.8 μg/g lung (d.w.)). Silver concentrations in the heart, liver, and brain were all below the detection limit. Assuming a minute volume of 25 ml/min, an aerosol concentration of 3.3 mg/m^3 ^and a deposition coefficient of 0.15 in the tracheo-bronchiolar and pulmonary region, we estimate that 803 μg Ag/g lung (d.w.) was delivered to the lungs. Thus, 4% of the nominal Ag dose was found in the pulmonary region.

### Bronchoalveolar Lavage Fluid

**Figure 5:**
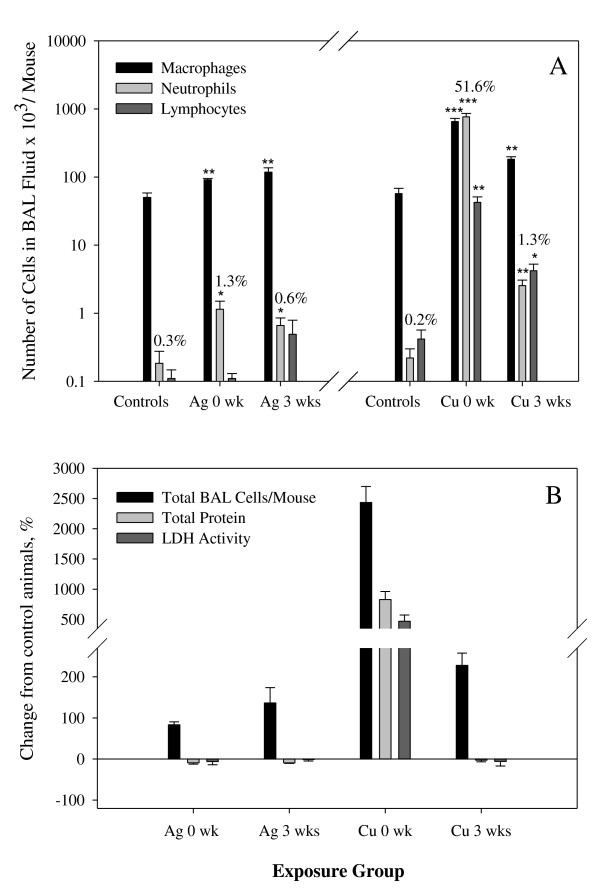
**Results from analysis of BAL fluid after sub-acute exposure to Ag nanoparticles (n = 5 per each group)**. Data from Ref. 34 for Cu nanoparticle exposure (n = 6 per control group, n = 8 per Cu-exposed group) using the same protocol are shown for comparison. Number and percentages of differential cells in BAL, * p < 0.05 and ** p < 0.01 (A). Percent change from control animals in total cells, total protein and activity of LDH (B). Data are expressed as mean ± SE.

The total number of cells per mouse in BAL fluid was significantly increased in Ag-exposed mice necropsied at 0 wk (92.3 ± 3.7 × 10^3^) and 3 wks (119.2 ± 18.7 × 10^3^) post exposure, compared to sham-exposed mice (50.1 ± 8.4 × 10^3^). As shown in Figure 5A, the number of neutrophils per mouse in BAL fluid was significantly increased in mice necropsied at 0 wk and 3 wks post exposure (p < 0.05). However, this change was of little biological significance. Additionally, there was no significant change in total cell numbers between mice necropsied at 0 wk and 3 wks post exposure (Figure 5B). These results are in contrast to the effects of exposure to 12 nm copper nanoparticles with a copper oxide coating using the same protocol and with a similar airborne concentration (3.68 mg/m^3^) [34]. Data for copper shown for comparison in Figures 5A and 5B indicate a substantial recruitment of cells to the lungs and a substantial increase in total protein leaking from the epithelium. These resolved considerably after 3 wks without exposure.

We did not find significant differences between groups in total protein levels and activity of LDH in BAL fluid (Figure 5B). Concentrations of the following cytokines measured in BAL fluid were below the limit of detection: IL-6, TNF-α, MCP-1, MIP-1α, GM-CSF (Table 2). We found slight, though statistically significant, elevation in IL-12(p40) and KC concentrations in the group necropsied at 0 wk post exposure compared with sham-exposed mice (p < 0.05). These concentrations were still increased, but not significantly in the mice necropsied 3 wks post exposure. In contrast, Cu nanoparticles induced substantial elevations in MCP-1, IL-12(p40), MIP-1α, KC and TNFα. IL-6 and GM-CSF were also significantly elevated (Table 2).

**Table 2:** Cytokine concentrations in BAL fluid from mice exposed to Ag nanoparticles by inhalation. Data from Ref. 34 for Cu nanoparticle exposure using the same protocol are shown for comparison.

Exposure	IL-6	IL-12(p40)	TNF-α	KC	MCP-1	MIP-1α	GM-CSF
Group							
Silver	pg/ml						

Controls	ND	29.1 ± 2.9	ND	ND	ND	ND	ND

Ag 0 wk post exposure	ND	64.1 ± 10.6*	ND	4.24 ± 0.88*	ND	ND	ND

Ag 3 wks post exposure	ND	53.6 ± 21.9	ND	2.96 ± 0.54	ND	ND	ND

Detection limit	0.67	0.73	44.3	1.77	9.58	27.31	1.48

Copper				pg/ml			

Controls	ND	35.0 ± 6.8	21.4 ± 3.3	0.90 ± 0.12	ND	ND	0.16 ± 0.08

Cu 0 wk post-exposure	82.8 ± 13.8*	1210 ± 10.1*	161 ± 10.4*	215 ± 17.0*	4400 ± 401*	309 ± 37.2*	70.2 ± 10.9*

Cu 3 wk post-exposure	ND	42.7 ± 4.8	17.3 ± 1.6	1.44 ± 0.12	ND	ND	0.27 ± 0.13

Detection limit	0.11	0.08	13	0.19	3.44	2.01	0.08

ND Not detectable

* Significantly different from sham-exposed animals (p < 0.05)

The mean concentrations of Ag ion in BAL supernatants were 13.9 ± 0.9 μg/L and 1.7 ± 0.2 μg/L in animals necropsied at 0 wk and 3 wks post exposure, respectively. Silver was not detectable in BAL fluid from control mice.

**Figure 6:**
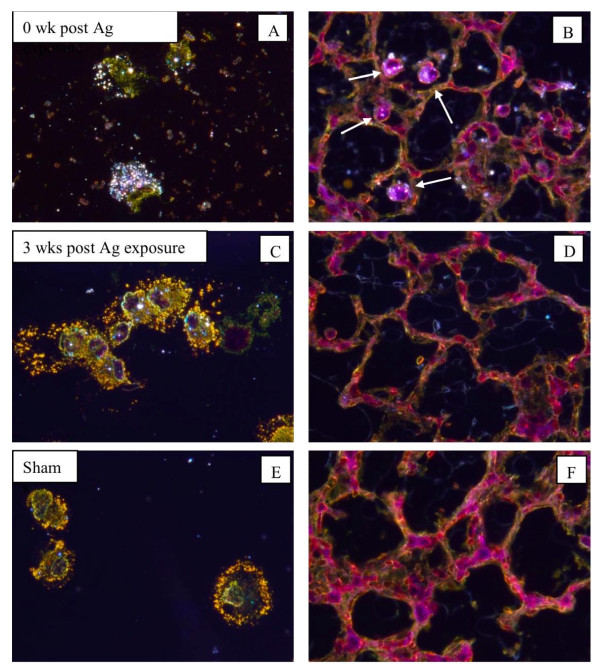
**Dark field micrographs of alveolar macrophages recovered from BAL fluid prepared by cytospinning and H & E staining (A, C, E) and lung tissues with H & E staining (B, D, F)**. Micrographs from animals exposed to nanosilver and necropsied immediately post exposure (A, B) or 3 weeks post exposure (C, D) and sham-exposed mice (E, F). White arrows point to particle-laden macrophages in lung tissue.

**Figure 7:**
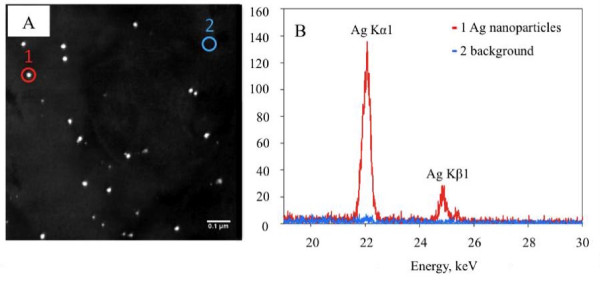
**Particles found inside alveolar macrophages were identified using TEM-EDS as silver (A, B)**.

No pathologic changes were found in Ag-exposed animals or sham-exposed mice. No signs of inflammatory cell infiltrate, alveolitis, perivasculitis, lymphoid agglomerates, epithelial damage, granulomas, giant cells or fibrosis were observed. Particle-laden macrophages were found in the BAL fluid of nanosilver-exposed mice as well as in the lung parenchyma immediately after the last exposure as shown using dark field microscopy (Figures 6A) and also by TEM-EDS of BAL macrophages (Figure 7A, B). Three weeks post exposure there was still evidence of silver particles in macrophage phagosomes (Figures 6C). However, there was a higher percentage of macrophages (76%) found in BAL fluid of mice necropsied at 0 wk post exposure as compared to mice necropsied 3 wks post exposure (28%).

## Discussion

The purpose of this study was to assess the biological response of mice to inhaled nanosilver and to determine the amount of silver retained in lungs and other organs. We observed nanosilver agglomerates in the inhalation chamber with a GM mobility diameter of 79 nm (Table 1) and SEM-EDS analysis showed the presence of Ag aerosols in the exposure chamber (Figure 4). ICP-OES analysis of nanosilver incubated in the simulated biological fluids revealed negligible formation of Ag ions (Figure 3). Moreover, most of the nanoparticles, even PVP coated particles, aggregated and settled out in these simulated biological fluids at the relatively high concentrations investigated after 24 hours and the XRD analysis of the precipitates revealed the presence of metallic Ag similar to Figure 2A.

Since there are limited data available on the concentrations of nanosilver in consumer products or in the air in occupational settings where materials with nanosilver are being produced, we established exposure concentrations in the whole-body chamber that were similar to our previous studies of toxicity assessment of titanium dioxide, copper, and iron. This facilitated comparison of toxicologic responses with our previous studies with different nanomaterials [34,41,42]. The median amount of silver measured in lungs of nanosilver-exposed mice was 31 and 10 μg/g lung (d.w.) for 0 wk and 3 wks animals, respectively, which corresponds to 100 μg/L and 32 μg/L Ag concentrations measured by ICP-OES in the lungs. Moreover, low Ag concentrations in the particle-free supernatants of BAL fluid (13.9 ± 0.9 μg/L and 1.7 ± 0.2 μg/L in animals necropsied at 0 wk and 3 wks post exposure, respectively) were detected by ICP-MS. Higher concentration of Ag measured in BAL fluid from animals necropsied at 0 wk agrees with the higher amount of Ag measured in the 0 wk lungs. Thus, these data suggest that there is some evidence for Ag dissolution in the lungs. It has been shown that Ag nanoparticles might fractionate (or dissolve) in aquatic environments at very low (< 100 μg/L) concentrations since nanoparticles might be less aggregated than in higher concentrated samples [43,44].

The amount of Ag detected in the 0 wk group corresponds to the lung burden accumulated by a 70 kg person exposed to a very high concentration of 1.0 mg/m^3 ^for 16.6 hours. Hence, this represents a substantial dose. To calculate the exposure time of the human, we assumed exposure concentration of 1.0 mg/m^3^, frequency of breathing 15 breaths/min, tidal volume of the lungs 600 mL/breath, pulmonary deposition fraction for 80 nm particles of 0.4 (based on human model by Cassee et al. [38]) and delivered Ag dose to human 3574 μg. The amount of nanosilver measured from the pulmonary region of the exposed mice was below the estimated levels based on the minute ventilation, deposition fraction and gravimetric measurements of chamber outflow. This could be due to lower breathing zone concentrations for the mice than expected from the chamber outflow measurements, an overestimation of the deposition coefficient for the pulmonary region, clearance by mucociliary escalator to the trachea or gastrointestinal tract, translocation of nanosilver to other organs that we did not measure, or poor sensitivity of the ICP-OES assay methods. Work by Asgharian and colleagues has shown that particles below 10 nm are deposited in the nasopharynx and tracheal regions with little penetration into the pulmonary region [37]. Deposition occurs primarily through axial transport by diffusion and convective mixing. Model predictions show that for nanoparticles in the 80 nm range, the deposition fraction for the pulmonary region is about 0.4 in humans (for mouth breathing) and 0.15 in rodents which are obligate nose breathers [38].

We observed increased total cell numbers in Ag-exposed animals (mainly macrophages) and a slight inflammatory response (increased number of neutrophils in BAL fluid) in 0 wk mice (Figures 5). Recruitment of macrophages is a normal clearance mechanism following particle exposure [45,46]. Neutrophilia decreased after 3 weeks without exposure, however total number of cells remained elevated. Interestingly, comparison of these results to other metal nanoparticle exposures studied by our group shows that nanosilver is most similar to iron nanoparticles, which are coated with an iron oxide layer. In the previous study, iron nanoparticles were found to be significantly less toxic than copper nanoparticles [34]. Copper nanoparticles induced a 25 times increase in the number of BAL cells after sub-acute exposure as well as an increase in most cytokines.

The levels of total protein and LDH activity in BAL fluid did not change significantly with exposure to nanosilver (Figure 5B). This is another indication of low nanosilver toxicity at the exposure concentration used in this study. Of 7 cytokines tested only two, IL-12(p40) and KC, had slightly elevated concentrations at 0 wk and most were below detection even using a very sensitive assay (Table 2). IL-12(p40), which exhibited the most elevated concentration, functions primarily to induce naïve T-cells to differentiate to Th_0 _cells and to enhance NK cell activity. It is produced by dendritic cells and macrophages [47]. This is consistent with the observed doubling in BAL macrophages but is very low in comparison to our previous study of Cu nanoparticles when we detected a mean level of IL-12(p40) of 1210 pg/ml (Table 2). Keratinocyte-derived cytokine (KC) that is involved in chemotaxis and cell activiation of neutrophils, was also slightly elevated. However, this increase was 50 times lower than in Cu-exposed mice. Furthermore, other chemokines/cytokines such as MCP-1, MIP-1α, TNF-α, RANTES, GM-CSF were much more elevated in case of Cu nanoparticle exposure [34].

We observed agglomerated nanosilver in macrophages that were recovered from BAL fluid and also in lung tissue (Figure 6). Dark-field microscopy revealed lower particle load at 3 wks post exposure than immediately post exposure, showing some particle clearance or possible translocation from the airways to the interstitium. Lung tissues were without remarkable pathological changes. In a 90 day rat study, lung inflammation and an increase in granulomatous lesions as well as alterations in lung functions were observed [48]. In another inhalation study in rats, minimal, toxicologically insignificant effects of nanosilver on the nasal respiratory mucosa were indicated [49].

Here we used the same experimental mouse model and exposure system as in our previous studies on the toxicity of different nanomaterials [34,41,42]. Of all nanomaterials studied in the size range from ca. 5 to 20 nm (TiO_2_, Fe, Cu, Ag), copper nanoparticles, which had a copper oxide coating, induced much higher total cell and neutrophil recruitment in BAL fluid, elevated total protein, activity of LDH and levels of inflammatory cytokines in BAL fluid than nanosilver [50]. This increased inflammatory response in copper-exposed mice was associated with the nanoparticle size and increased ion concentration produced from the dissolving nanoparticles *in vivo*. Mice exposed sub-acutely to 2-5 nm TiO_2 _nanoparticles showed a moderate inflammatory response at 0, 1, and 2 weeks post-exposure that resolved after 3 weeks post-exposure. Iron nanoparticles induced a minimal inflammatory response similar to nanosilver. The toxicity of Ag nanoparticles in this murine inhalation model was similar to Fe nanoparticles. Both types do not dissolve in artificial interstitial fluid which could be associated with the negligible toxic response observed for metal and metal oxide nanomaterials. Other studies also support a role for solubility of nanomaterials in their toxicity [51,52].

## Conclusions

The toxicity of nanosilver is a highly debated topic in the literature and in reports of the U.S. Environmental Protection Agency. Our study indicates that 40 hr inhalation of 3.3 mg/m^3 ^nanosilver induced minimal pulmonary toxicity or inflammation. Methods used to evaluate toxic responses in this study indicate much lower inflammatory response of mice to silver nanoparticles than to copper nanoparticles. These observations are in agreement with other studies, which indicate that much higher exposure doses of silver nanoparticles than other metals are needed to induce significant inflammation.

## Competing interests

Vicki H. Grassian is a consultant and a member of the scientific advisory board of Vive Nano (Toronto, Canada) and has stock shares in Nanoscale Corp (Manhattan, Kansas).

## Authors' contributions

LVS participated in the nanoparticle characterization, conducted dissolution study and analysis of elements in mice organs, drafted parts of the manuscript and integrated the text into a complete draft; AAD drafted parts of the manuscript related to the toxicity results, conducted the mouse exposure studies, BAL evaluation and histopathological analyses; JSK conducted the mouse exposures, BAL evaluation, histopathology results analysis and ICP-OES organ preparation and analyses; HP participated in the nanoparticle characterization and in ICP-OES analysis of mice organs; PTO led the design of the exposure and generation system, and coordinated the exposure assessment and aerosol characterization; VHG designed the integrated study, participated in its coordination and completion including analysis of nanoparticle characterization data, manuscript outlining and writing, and editing of final manuscript; PST designed and coordinated all aspects of the toxicity study including experimental design and analysis and authored the final manuscript. All authors read and approved the final manuscript.
